# Modulation of Hydrogen Peroxide Production in Cellular Systems by Low Level Magnetic Fields

**DOI:** 10.1371/journal.pone.0022753

**Published:** 2011-08-26

**Authors:** Carlos F. Martino, Pablo R. Castello

**Affiliations:** 1 Electrical, Computer, and Energy Department, University of Colorado Boulder, Boulder, Colorado, United States of America; 2 Molecular, Cellular, and Developmental Biology, University of Colorado Boulder, Boulder, Colorado, United States of America; Mizoram University, India

## Abstract

Increased generation of reactive oxygen species (ROS) and an altered redox status have long been observed in cancer cells, suggesting that ROS might be involved in the development of these cells. However, recent studies suggest that inducing an excess of ROS in cancer cells can be exploited for therapeutic benefits. Cancer cells in advanced stage tumors frequently exhibit multiple genetic alterations and high oxidative stress, suggesting that it might be possible to preferentially modulate the development of these cells by controlling their ROS production. Low levels of ROS are also important for the development and survival of normal cells. In this manuscript, we present data on the influence of the suppression of the Earth's magnetic field (low level magnetic fields or LLF) which magnitudes range from 0.2 µT to 2 µT on the modulation of hydrogen peroxide (H_2_O_2_) in human fibrosarcoma cancer cell line HT1080, pancreatic AsPC-1 cancer cell line, and bovine pulmonary artery endothelial cells (PAEC) exposed to geomagnetic field (control; 45 µT–60 µT). Reduction of the Earth's magnetic field suppressed H_2_O_2_ production in cancer cells and PAEC. The addition of catalase and superoxide dismutase (SOD) mimetic MnTBAP inhibited the magnetic field effect. Modulating ROS production by magnetic fields may open new venues of biomedical research and therapeutic strategies.

## Introduction

Reactive oxygen species (ROS) specially de superoxide anion (O_2_^.−^) are short-lived species with half-lives of less than a nanosecond to several seconds [1] and are a consequence of the incomplete reduction of dioxygen by several system including the complex I and III of the mitochondrial respiratory chain, the enzyme Xantine oxidase, etc. They are linked to normal cellular events such as signaling, regulated growth, proliferation and programmed cell death, and have also been associated with certain forms of cell and tissue pathology [2], [3]. During normal metabolic activity cells remain in a homeostatic state whereby their rate of ROS production is kept in balance through redox regulatory mechanisms that utilize several systems of antioxidant scavenging [4]. ROS are subdivided into O_2_^.−^, H_2_O_2_, hydroxyl radicals (OH^.^) and singlet oxygen, molecules, and their source of production include the mitochondria, endoplasmic reticulum, plasma membrane and cytosol. ROS are a necessary biochemical component for the maintenance of healthy cells and tissues, however, in excess concentration they can be highly cytotoxic leading to cell and tissue damage [5]. For example, in comparison to normal healthy cells, ROS have been implicated in the onset and progression of certain cancer types with the observation that cancer cells in particular demonstrate an increased synthesis of O_2_^.−^ [6]. This can be attributed to the increased oxidative stress load associated with increased metabolic activity, particularly at the level of the mitochondria, to sustain the increased rate of cell division, which is the hallmark of cancer. However, in order to maintain a healthy balance of intracellular ROS, organisms are equipped with the antioxidant enzymes: SOD, glutathione peroxidase (GPx), catalase, and thioredoxin reductase (TPx), which reduce these potentially cytotoxic molecules to a less harmful form [4].

O_2_^.−^ is perhaps one of the most important free radicals in biology. This radical comes from the incomplete reduction of oxygen and several known enzymes catalyze it. O_2_^.−^ contains an unpaired electron that makes it highly reactive and likely to be affected by the magnetic fields [7]. Despite its high reactivity, O_2_^.−^ is rapidly decomposed by SOD to form H_2_O_2_. The H_2_O_2_ can then be decomposed by catalase, GPx and TPx. H_2_O_2_ can also produce the highly reactive OH^.^ in the presence of the Fe^2+^cation (Fenton reaction), and both OH^.^ and O_2_^.−^ can react with other molecules to form new radicals such as peroxides [8], [9]. Given the fact that both H_2_O_2_ and O_2_^.−^ can react with any kind of biological macromolecule, and their concentration is dependent on both, production and clearance, ROS homeostatic balance is a key factor in the regulation of biological processes that they control [1].

In our previous papers, we presented results on the inhibition of cancer cell growth rate by reducing the Earth magnetic field [10] and the response and function of endothelial cells to these LLF [11]. In both experimental models, LLF effects were compared to the geomagnetic field (45 µT–60 µT). A possible mechanism of interaction between the biological systems and the magnetic fields is a process where free radical may be involved [12]–[14]. This mechanism has been suggested to be able to occur even for magnetic fields of environmental intensities [15], [16], and the observed low-level magnetic field effects by our group may be predicted by this model [17].

In this study we have investigated a mechanism by which LLF may modulate reactive oxygen species (ROS) production in cells thereby affecting their growth, proliferation and survival rate [18]. Thus, modulating ROS production by magnetic fields may open new venues of biomedical research and therapeutic strategies including the role of ROS in certain aspects of tumor cell angiogenesis, programmed cell death [19], [20], and possible cell regeneration.

The purpose of this study was to examine the role that LLF plays on ROS production in cancer cells lines, primary endothelial cells, and pancreatic cells. This was achieved by measuring rates of H_2_O_2_ production using fluorometric methods. H_2_O_2_ can freely pass through cell membranes and can be detected outside the cell. The extracellular quantification of the production of H_2_O_2_ may be a direct measure of the production and decomposition of O_2_^.−^ [21], [22]. For the quantification of H_2_O_2_ we will use a method that has been successfully applied before [23]. This method involves the oxidation of Amplex Red by H_2_O_2_ in the presence of horseradish peroxidase (HRP) to produce the fluorescent product resorufin.

## Materials and Methods

### Cell Culture

Fibrosarcoma HT1080 and pancreatic AsPC-1 cancer cells (ATCC #CCL-121, #CCL-1682, Manassas, VA) were grown and maintained in EMEM (Eagle's Minimum Essential Medium) and RPMI 1640, respectively, supplemented by 10% Fetal Bovine Serum (ATCC, Manassas, VA). Bovine PAECs were maintained in a growth medium (D-Valine MEM medium) containing 10% Fetal Bovine Serum, 2% *L*-glutamine (Invitrogen). The cells were cultured in 75 cm^2^ flasks to expand cell number (Life Science Products, CO). After reaching confluence, the cells were seeded in 35 mm Petri dishes (BD Falcon). The volume of medium totaled 2 ml. Medium was then changed every two days. The cultures were incubated in a 5% CO_2_ atmosphere at 37°C in the same incubator (Fisher Scientific, Model 5). The temperature and CO_2_ levels were monitored daily and maintained at 37°C and 5%, respectively. The difference in temperature between the upper and lower areas of the incubator was 0.1°C (0.003°C/cm). All experiments were conducted in the same incubator. To control for location in the incubator and any associated electromagnetic noise or other spatial variation, the orientation of experimental and control cultures were periodically reversed. Cells were seeded and allowed to rest for twenty-four hours under the same magnetic background conditions, at which timed magnetic exposures began. This time is denoted as t_0_.

### Magnetic Stimulating System

The background static magnetic field intensity inside the incubator used in these studies varied considerably as measured by a gauss meter (Walker Scientific, Model FGM 4D2N), which depends on the relative position in the room and the relative position of surrounding objects. The control group in this report is subjected to 45 µT static fields as follows. A tri-axial square Helmholtz coil consisting of 25 turns and 12.5 cm on each side separated by 6.25 cm implemented a uniform 45 µT static magnetic field perpendicular to the plane of growth of the cells (z-direction; x–y fields were canceled). The coils were driven by a power supply (HP 6205C Dual, Hewlett Packard, Palo Alto, CA) and resistive circuitry.

Static LLF were implemented by shielding the fields with μ-metal cylinder. In the μ-metal cylinder, the static fields inside registered between 0.5 µT–2 µT. Temperature measurements were made inside the cylinder and in the coils; the temperature difference was 0.1°C, which is consistent with the variation inside the incubator.

The background time-varying magnetic field was measured by induction with a sensor comprised of a set of square mutually perpendicular coils (Diameter 1 = 9.92×10^−04^ m, Diameter 2 = 9.70×10^−04^ m, Diameter 3 = 7.56×10^−04^ m, each with a thickness of 2×10^−03^ m and 200 turns, fed in twisted pair). The signal from the coils was amplified and filtered (10 KHz low pass filter) by a high impedance differential amplifier (OSP-1 Oscilloscope Preamplifier, Advanced Research instruments, Golden, CO, USA) and the spectrum up to 600 Hz was analyzed. The complete time-varying sensing system inherent noise was below 2.41×10^−06^ T*Hz. The field measurements obtained for each experimental environment are presented in Table 1.

**Table 1:** The magnitudes reported for time-varying (AC) measurements were obtained by vector summation of the fields recorded at the 3 perpendicular axes (x,y,z).

Background AC Magnetic Field (up to 600 Hz)					
		Environment tested	Dominant Frequency (Hz)	Amplitude (T)	Attenuation(dB)
**Incubator**	**Top Shelf**	Max measured	60	3.67E-06	−20.98
		Min Measured	60	1.63E-06	−17.46
		Max measured (inside μ-metal)	60	1.62E-06	−17.43
	**Bottom Shelf**	Max measured	60	3.86E-05	−31.20
		Min Measured	60	2.67E-06	−19.61
		Max measured (inside μ-metal)	60	6.08E-06	−23.17

The measurements reported for each environment correspond to the frequency with maximum amplitude in a spectrum of up to 600 Hz. All spectrums had maximums at 60 Hz and they were mainly composed harmonics of that frequency. The attenuation values are referenced to the average of readings made on the center of the room with no nearby electricity consuming devices.

### Fluorometric Detection of H_2_O_2_ Production

H_2_O_2_ was measured using the horseradish peroxidase-linked Amplex Ultra Red™ (Invitrogen) fluorometric assay. Fibrosarcoma cells and endothelial cells were seeded at a concentration of 2.0×10^5^ cells per 35 mm Petri dish and allowed to rest for 24 hours prior to experiment. Medium was aspirated and 2 ml of new medium was added containing 0.2 units/ml horseradish peroxidase and 100 µM Amplex UltraRed (AUR). By using these measurement conditions we were able to keep the linear response of the AUR system for incubation times longer than 36 hours. Resorufin fluorescence was followed by a Gemini fluorescence microplate reader (Molecular Devices, Sunnyvale, CA). H_2_O_2_ calibration curves with HRP-AUR in LLF do not show any difference compared to control, thus demonstrating that LLF do not interact with the detection system.

### Statistical Analysis

Statistical analysis was performed using 1-way ANOVA with a minimal confidence level of 0.05 for statistical significance. Each experiment in vitro was performed at least 3 times with a minimum of 3 samples per termination point, resulting in a total number of 6 samples for each experiment. The data shown constitutes a representative sample of the experiments performed.

## Results

### LLF suppress H_2_O_2_ production in fibrosarcoma cancer cells

**Figure 1:**
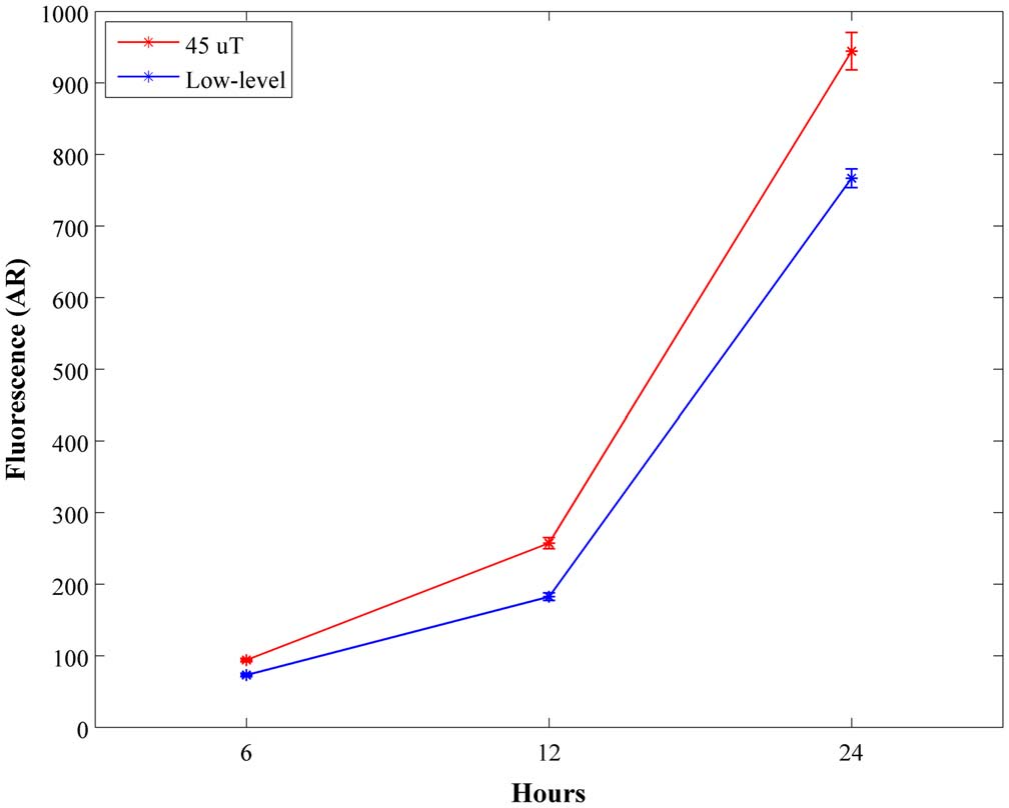
LLF decrease H_2_O_2_ production in HT1080 cancer cells (p<0.05). H_2_O_2_ production increased through out the exposure time. Low level fields decreased levels of H_2_O_2_ production in fibrosarcoma cells over 25% during the 24 hour exposure time.

H_2_O_2_ was measured using the horseradish peroxidase-linked Amplex Ultra Red™ fluorometric assay (Invitrogen). LLF decrease ROS activity in fibrosarcoma cancer cells (Fig. 1; p<0.05). Levels of ROS activity decreased 30%, 25%, and 25% after 6 hr, 12 hr, and 24 hr exposure to LLF respectively. Catalase was added as control at a concentration of 40 units/ml. Addition of external catalase suppressed the geomagnetic field effects on H_2_O_2_ production; catalase brought GMF levels of H_2_O_2_ production to LLF levels (data not shown).

### Effect of SOD mimetic on H_2_O_2_ production in fibrosarcoma cells

**Figure 2:**
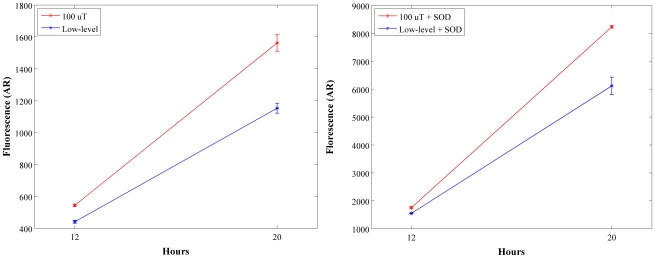
Modulation of LLF effect by SOD mimetic. *Left panel*: production of H_2_O_2_ in HT1080 cancer cells (p<0.05) in the presence (100 µT) or in the absence (low level) of magnetic field. *Right panel*: Effect of the SOD mimetic MnTBAP on the H_2_O_2_ production induced by magnetic fields.

In order to verify that the H_2_O_2_ production in this particular cell line is dependent on the dismutation of O_2_^.−^ anion by SOD, in a separate control, we assayed the effect of the magnetic fields in the presence of the ROS scavenger Manganese (III) tetrakis (4-benzoic acid) porphyrin chloride (MnTBAP) (Fig. 2, right panel). Adding a 200 nM concentration of MnTBAP to the cell medium did not alter the magnetic field's final effect on decreasing the H_2_O_2_ production. However, when 2 mM of MnTBAP was added to the cell medium, the level of H_2_O_2_ in the unexposed sample increased to a level identical to the levels observed as a result of magnetic field exposure (data not shown). In other words, production of H_2_O_2_ was identical between LLF and control by the addition of 2 mM MnTBAP. Also, the difference of H_2_O_2_ production of HT1080 cells exposed to 100 µT (Fig. 2, left panel) is greater than HT1080 cells exposed to 45 µT when normalized to each proper LLF group. These results combined suggest a dose dependence of H_2_O_2_ production on magnetic field intensity and MnTBAP concentration.

### H_2_O_2_ production in pancreatic cancer cell become indistinguishable between LLF and geomagnetic field

Next we proceeded to investigate if H_2_O_2_ production differs between distinct cancer cells subjected to LLF. The same protocol as the previous experiment for HT1080 was used. Differences in H_2_O_2_ production become indistinguishable after 12 hr and 24 hr between the LLF and the control for pancreatic cancer line AsPC-1 (data not shown).

### LLF also inhibits H_2_O_2_ production in endothelial cells

**Figure 3:**
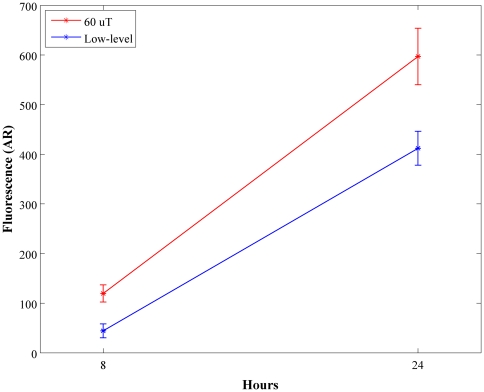
Low level fields decreased H_2_O_2_ production in endothelial cells.

In view of the last results, production of H_2_O_2_ may differ between cancer cells and primary cells. Previous results showed that LLF inhibit growth rates of endothelial cells [11], which may be related to the suppression of H_2_O_2_. Specifically, LLF suppressed hydrogen peroxide production in endothelial cells after 8 hours and 24 hours of exposure (Fig. 3; p<0.05).

## Discussion

Targets for oxidative processes are molecular complexes that readily give up or acquire a single electron. The redox reactions, with simple transfer of electrons affect almost all complex biological processes and have profound effects on cell growth, proliferation, survival and the propagation of various pathological processes, to include cancer.

We have shown that exposure of fibrosarcoma cancer cells and primary endothelial cells to low level magnetic fields modulate H_2_O_2_ production and that the addition of free radical scavengers suppressed this magnetic field effect. Previous results on inhibition of growth rates of cancer cells and endothelial cells by reducing the Earth's magnetic field suggest that changes in H_2_O_2_ production may be a consequence of the magnetic field effect [10], [11]. However, it is unknown whether the inhibition of growth observed is a result of biologic events that may involve cell cycle modulation, cell signaling or possibly the induction of apoptosis [10].

We suggest here that the magnetic sensitivity of cellular systems may involve superoxide. In recent reports, it has been hypothesized that superoxide radical may be involved in the magnetoreception of birds [24], [25]. Low level fields may modulate the interconversion of singlet to triplet product yields involving the superoxide free radical [25], [26]. Inhibition of growth rates by low level fields [10] together with suppression of H_2_O_2_ production provide evidence supporting the hypothesis presented in the introduction.

Singlet (S; antiparallel electron spins) and triplet (T; parallel electron spins) lie at the heart of the radical pair mechanism. There are a number of conditions to be met for a radical pair to respond to an external magnetic field [27], [28]: a) the singlet and triplet states must have different chemical fates; b) the reactivity of the pair should be spin dependent; c) and magnetic hyperfine interactions should be present in one or both radicals, which drives the oscillations 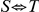. The interconversion is also influenced by the interaction of the electron's spin and the magnetic field, which is known as the Zeeman interaction. Both radical partners contribute to the hyperfine interaction of the radical pair. Most organic radicals have individual coupling constants ranging from 1–10 G [25], [28]. A free radical pair involving superoxide, with nuclear spin state zero, decreases the hyperfine interaction. In order to observe changes in reaction yields in the presence of the Earth's magnetic field, the hyperfine interaction between radical pairs should be in the same order of magnitude as the Zeeman interaction [25], [26], [28], thus making the possibility for the magnetic field effect.

Reducing the Earth's magnetic field inhibits H_2_O_2_ production in cellular systems. Suppression of growth rates of cancer and primary cells by LLF, as we previously reported [10], [11], may be linked to the modulation of H_2_O_2_ by magnetic fields. The addition of catalase suppress the increment of ROS production by its direct role in the decomposition of H_2_O_2_. Nevertheless, the effect of MnTBAP suppressing the effect of the normal magnetic field suggests that O_2_^.−^ dimutation by SOD would be a possible point of intervention of the magnetic field with this particular system. The biological system and the relative change of H_2_O_2_ production at distinct static magnetic field intensity points out to a free radical mechanism. In this study we show that not all the cells types may share the same mechanism of ROS control by magnetic fields. Pancreatic cell are a clear example of that. Interestingly, most cancer cells undergo increased oxidative stress and ROS production due to excessive metabolic requirements to support an increased rate of cell division. It is reasonable to consider investigating a therapeutic that might target cancer cells through a free radical-mediated mechanism. Whether radio frequency exposure can influence growth rates and H_2_O_2_ production remains open and we propose to investigate it in future studies.
